# Reduction in complication rate and hospital length of stay following robotic arm-assisted simultaneous bilateral total knee arthroplasty when compared to conventional techniques: a cohort study with minimum one year follow-up

**DOI:** 10.1007/s11701-026-03226-7

**Published:** 2026-02-23

**Authors:** William Jordan, Tim Cheok, Kristopher Law, Julie F. Vermeir, Anthony M. Silva

**Affiliations:** 1 Department of Orthopaedic Surgery, The Prince Charles Hospital, Chermside, QLD Australia; 2 College of Medicine and Public Health, Flinders University, Bedford Park, South Australia Australia; 3 Graduate School of Medical Sciences, University of Groningen, Groningen, The Netherlands; 4 Department of Orthopaedic Surgery, St Vincent’s Private Hospital Northside, Chermside, QLD Australia; 5 University of Queensland Centre for Clinical Research (UQCCR), Faculty of Medicine, The University of Queensland, Herston, QLD Australia; 6 Faculty of Medicine, The University of Queensland, Herston, QLD Australia

**Keywords:** Bilateral simultaneous knee arthroplasty, Robotic, Conventional, Mortality, Morbidity, Patient reported outcomes

## Abstract

Bilateral simultaneous total knee arthroplasty (TKA) may be indicated in patients with symptomatic bilateral knee arthritis; however, it is associated with higher morbidity and mortality risk compared to staged procedures. We retrospectively reviewed prospectively collected data comparing bilateral simultaneous robotic arm-assisted TKA (RA-TKA) with conventional TKA (CO-TKA), with a minimum 12-month follow-up period. The primary outcomes of interest were 12-month mortality, revision and reoperation rates, as well as inpatient complication rates. Secondary outcomes of interest were surgical duration, hospital length of stay (HLOS), and functional outcomes after 12 months. The functional outcomes were quantified using the Oxford Knee Score (OKS), EQ-5D-5L index, and EQ-VAS. Each outcome was adjusted for relevant patient and surgical factors. A total of 93 patients were identified for inclusion – 50 in the CO-TKA group and 43 in the RA-TKA group. None of the patients in our cohort died within 12 months of undergoing TKR, nor did any require revision surgery during the follow-up period. There was no difference in reoperation rates (hazard ratio = 1.36; p = 0.877); however, there was a significant decrease in the odds of inpatient complications (odds ratio = 0.21, p = 0.023). There was no difference in surgical duration (p = 0.849), but HLOS was shorter in the RA-TKA group by 1.1 days (p = 0.041). Although we found a statistically significant improvement in the OKS by 2.3 points (p = 0.041) in the RA-TKA group, this was not clinically relevant. There was also no difference in EQ-5D-5L index between the two groups (p = 0.335); however, the RA-TKA group demonstrated a clinically and statistically significant increase in EQ-VAS by 10.8 points (p = 0.037). In our patient cohort, bilateral simultaneous RA-TKA was associated with reduced HLOS and lower complication rates, with no substantial differences in other outcomes except for a significant improvement in the EQ-VAS score.

## Introduction

Total knee arthroplasty (TKA) is the standard of care for the management of symptomatic end-stage osteoarthritis of the knee [1]. Approximately a quarter of patients presenting with symptomatic knee osteoarthritis have bilateral disease, and of those with unilateral disease, 80% would develop contralateral osteoarthritis within a decade [2]. In patients presenting with symptomatic bilateral knee osteoarthritis refractory to conservative management, controversies remain as to whether TKA should be performed simultaneously or in a staged manner. Simultaneous bilateral knee arthroplasty has been previously thought to carry a significant risk of morbidity and mortality, even in healthy patients [3–5]. Nonetheless, this strategy is associated with a shorter rehabilitation period and reduced healthcare expenditure. Furthermore, a recent study by Kim et al. found no difference in the safety profile between bilateral simultaneous, staggered and staged TKA [6].

Robotic arm-assisted TKA (RA-TKA) has been shown to improve component positioning and minimise soft tissue trauma, compared to conventional TKA (CO-TKA) [7–10]. RA-TKA has been shown to reduce hospital length of stay (HLOS) and functional outcomes in patients undergoing unilateral TKA [11–13]. However, there is a paucity of literature surrounding the efficacy of bilateral simultaneous RA-TKA compared with CO-TKA. Cohen et al. found that there was no effect on 30-day mortality in patients undergoing bilateral simultaneous RA-TKA; however, HLOS and complication rates were lower compared to those undergoing CO-TKA [14]. A further study by Albelooshi et al. demonstrated no difference in functional outcomes following bilateral simultaneous RA-TKA compared with CO-TKA, but the former resulted in more predictable implant placement [15]. Importantly, Masilamani demonstrated that operating room efficiency increased when bilateral simultaneous TKAs were performed under robotic assistance [16].

The aim of this study was to compare the mortality, morbidity and outcomes following bilateral simultaneous RA-TKA with CO-TKA in patients followed up for a minimum of 12 months. Our primary outcome of interest was to compare all-cause mortality and associated morbidity between these two groups. Secondary outcomes of interest were to compare the surgical duration, HLOS, and functional outcomes in patients undergoing bilateral simultaneous RA-TKA versus CO-TKA.

## Materials and methods

### Patient selection and surgical intervention

This retrospective review of prospectively collected data was performed in a tertiary-level university teaching hospital located in Brisbane, Australia. Ethical approval was sought and obtained prior to the commencement of this study (Approval no.: AM/2025/MNH/113088). The decision to pursue bilateral simultaneous TKA was made following a collaborative discussion between the orthopaedic surgeon and patient, balancing potential risks against the anticipated benefits. Consecutive patients who underwent a bilateral simultaneous TKA at our institution between January 2009 and June 2024, with a minimum follow-up period of 12 months, were identified for potential inclusion. To reduce heterogeneity between the groups, we excluded those who did not receive a TKA using the Stryker Triathlon Total Knee System (Stryker, Michigan, USA). All TKAs were performed under the supervision of, or by a fellowship-trained consultant orthopaedic surgeon. Alignment technique, tourniquet use, and patella resurfacing were left to the discretion of the operating surgeon. All of the surgeons in our institution favoured patella resurfacing, although itwas not performed when anatomical limitations, such as having a thin (≤ 14 mm) or small patella, would have exposed patients to unnecessary risks [17–19]. In both groups, both knees were prepped and draped at the same time; however, the surgery started on one side first before moving to the contralateral side. Where RA-TKA was performed, we utilised the Mako Robotic Arm System (Stryker, Michigan, USA). In this group, the same robot was used for both sides, with the robotic system re-draped in a sterile fashion between sides. Turnaround time for the robot was not recorded.

Utilising our inclusion and exclusion criteria, we identified 93 patients for inclusion in our analysis – 50 patients (100 knees) in the CO-TKA group and 43 patients (86 knees) in the RA-TKA group. Median follow-up was 8.2 years (6.5–11.7) in the CO-TKA group and 3.2 years (2.0–3.8) in the RA-TKA group. There was no significant difference in age at the time of surgery (*p* = 0.744), sex (*p* = 0.675), body mass index (BMI) (*p* = 0.692), American Society of Anesthesiologists (ASA) classification (*p* = 0.090), smoking status (*p* = 0.659), Charlson Comorbidity Index (CCI) score (*p* = 0.935), or primary surgeon grade (*p* = 0.141) between the two groups. In terms of comorbidities, there was a significantly greater number of patients with depression/ anxiety (*p* = 0.040) in the RA-TKA group compared to the CO-TKA group. There were no differences in the proportion of patients with a history of cardiac arrhythmia (*p* = 1.000), coronary artery disease (*p* = 0.621), cardiac failure (*p* = 1.000), chronic obstructive pulmonary disease (*p* = 0.246), cerebrovascular disease (*p* = 1.000), chronic kidney disease (*p* = 1.000), diabetes (*p* = 0.700), hypertension (*p* = 0.385), chronic liver disease (*p* = 1.000) or peripheral vascular disease (*p* = 1.000) between the two groups. A greater proportion of patients in the RA-TKA group underwent the surgery without tourniquet use (*p* < 0.001) and had their patella resurfaced (*p* = 0.001). A detailed analysis of these covariates is displayed in Table 1.

**Table 1:** Demographics, comorbidities and surgical Factors. RA-TKA = Robotic arm-assisted total knee arthroplasty; CO-TKA = Conventional total knee arthroplasty; ASA = American society of anesthesiologists

Variable	CO-TKA (*N* = 50)	RA-TKA (*N* = 43)	*p*-Value
Age at surgery, years	63.0 (60.0–67.0)	63.0 (59.0–68.0)	0.744
**Sex**			0.675
Men	27 (54.0)	26 (60.5)	
Women	23 (46.0)	17 (39.5)	
Body Mass Index (kg/m²)	30.9 (28.1–34.6)	30.8 (27.4–34.4)	0.692
**ASA Classification**			0.090
1	10 (20.0)	2 (4.7)	
2	32 (64.0)	34 (79.1)	
3	8 (16.0)	7 (16.3)	
**Comorbidities**			
Cardiac Arrhythmia	1 (2.0)	0 (0)	1.000
Coronary Artery Disease	3 (6.0)	1 (2.3)	0.621
Cardiac Failure	0 (0)	0 (0)	1.000
Chronic Obstructive Pulmonary Disease	3 (6.0)	0 (0)	0.246
Cerebrovascular Disease	1 (2.0)	1 (2.3)	1.000
Chronic Kidney Disease	1 (2.0)	1 (2.3)	1.000
Depression/Anxiety	2 (4.0)	8 (18.6)	**0.040**
Diabetes	3 (6.0)	4 (9.3)	0.700
Hypertension	15 (30.0)	17 (39.5)	0.385
Chronic Liver Disease	0 (0)	0 (0)	1.000
Peripheral Vascular Disease	0 (0)	0 (0)	1.000
Active Smoker	2 (4.0)	3 (7.0)	0.659
Charlson Comorbidity Index Score	2.0 (2.0–3.0)	2.0 (2.0–3.0)	0.935
**Primary Surgeon Grade**			0.141
Consultant	30 (60.0)	21 (48.8)	
Fellow	7 (14.0)	3 (6.7)	
Resident	13 (26.0)	19 (44.2)	
**Tourniquet Use**			**< 0.001**
No	10 (20.0)	19 (44.2)	
Intermittent	15 (30.0)	12 (27.9)	
Released Prior to Closure	6 (12.0)	11 (25.6)	
Released at End of Case	19 (38.0)	1 (2.3)	
Patella Resurfaced	32 (64.0)	40 (93.2)	**0.001**

## Outcome measurement and analysis

The primary outcome of this study was to compare mortality and morbidity between those undergoing bilateral simultaneous RA-TKA and CO-TKA. The odds of all-cause mortality within 12 months from TKA was measured. Morbidity was assessed using revision and reoperation as endpoints, as well as the odds of inpatient complications. While conducting the survival analyses, patients were censored at death, or in the case of survival, on the 31st of June 2025. Differences between the two arms were then quantified using hazard ratios (HR) derived from a Cox-regression analysis. Furthermore, the difference in incidence of inpatient complications was quantified using odds ratios (OR). Complication was defined as those with a Clavien-Dindo grade 2 or above [20]. Each primary outcome was adjusted for age, sex, BMI, ASA classification, CCI score, primary surgeon grade, tourniquet use and patella resurfacing, where possible.

The secondary outcomes were to compare the surgical duration, HLOS, and functional outcomes in patients between the two groups. Surgical duration and HLOS were also adjusted for age, sex, BMI, ASA classification, CCI score, primary surgeon grade, tourniquet use and patella resurfacing to further account for differences in patient factors, surgeon experience, and surgical technique. We measured functional outcomes using the Oxford Knee Score (OKS) [21], EQ-5D-5L index and EQ-VAS score  after 12 months following their TKA [22]. These scores have previously been validated for use within the Australian knee arthroplasty population [23], and were adjusted for age, sex, BMI, ASA classification, CCI score, primary surgeon grade, and patella resurfacing.

Continuous variables were expressed as median (interquartile range) and compared using Mann-Whitney U test, whereas discrete variables were expressed as count (percentage) and compared using Fisher’s exact test. All statistical analyses were performed using Stata Version 18.0 (StataCorp LLC, Texas, USA). The threshold for statistical significance was set at *p* < 0.050.

## Results

### Mortality and morbidity

None of the patients in our cohort experienced mortality within 12 months, nor did they require revision surgery at any point during the follow-up period following their TKA. Adjusted analyses of these two outcomes were thus not possible. Four knees in three patients were reoperated on during the follow-up period – three from the CO-TKA group and one from the RA-TKA. The indication for reoperation in the CO-TKA group were stiffness requiring manipulation under anaesthesia (two knees in the same patient), and an arthroscopic biopsy to rule out infection in the remaining patient. Similarly, the reoperation in the RA-TKA group was for stiffness requiring manipulation under anaesthesia. Adjusting for age, sex, BMI, ASA classification, CCI score, primary surgeon grade, tourniquet use and patella resurfacing, there was no difference in reoperation risk between the two groups (HR = 1.36; *p* = 0.877).

The complication rate was 38.0% (19/50) for patients in the CO-TKA group and 23.3% (10/43) for patients in the RA-TKA group. A detailed breakdown of medical and surgical complications encountered is shown in Table 2. Of interest, the frequency of post-operative anaemia requiring blood transfusion and venous thromboembolic events were substantially greater in the CO-TKA group. The odds of any complication were significantly lower in the RA-TKA group compared to the CO-TKA group when adjusted for age, sex, BMI, ASA classification, CCI score, primary surgeon grade, tourniquet use and patella resurfacing (OR = 0.21; *p* = 0.023).

**Table 2:** Summary of Complications. Note that patients may have more than one complication. RA-TKA = Robotic arm-assisted total knee arthroplasty; CO-TKA = Conventional total knee arthroplasty; 95% CI = 95% confidence Interval; OR = Odds ratio

Complication	CO-TKA (*N* = 50)	RA-TKA (*N* = 43)	Adjusted analysis
All complications	19 (38.0)	10 (23.3)	OR = 0.21; 95% CI = 0.06–0.81; *p* = 0.023
*Surgical*	6 (12.0)	2 (4.7)	
Broken guide pin	0	1	
Intraoperative Fracture	1	0	
Latrogenic Injury to Medial Collateral Ligament	1	0	
Stiffness Requiring Manipulation Under Anaesthesia	1	1	
Superficial Surgical Site Infection	3	0	
*Medical*	16 (32.0)	8 (18.6)	
Cardiac Arrhythmia	3	0	
Anaemia requiring Blood Transfusion	7	2	
Severe Acute Kidney Injury	1	3	
Venous Thromboembolic Event	3	0	
Hospital Acquired Pneumonia	1	2	
Urinary Tract Infection	3	2	

## Surgical duration and HLOS

Median surgical duration was 179.0 min (142.0–239.0) in the CO-TKA group and 195.0 min (175.0–217.0) in the RA-TKA group. Adjusting for age, sex, BMI, ASA classification, CCI score, primary surgeon grade, tourniquet use and patella resurfacing, there was no difference in surgical duration between the two groups (*p* = 0.849).

Median HLOS was 7.0 days (6.0–8.0) in the CO-TKA group and 6.0 days (4.0–7.0) in the RA-TKA group. This was significantly shorter in the RA-TKA group compared to the CO-TKA group by 1.1 days when adjusted for age, sex, BMI, ASA classification, CCI score, primary surgeon grade, tourniquet use and patella resurfacing (*p* = 0.041).

### Functional outcome after 12 months

Median OKS was 46.0 points (43.0–47.0) in the CO-TKA group and 46.0 points (43.0–48.0) in the RA-TKA group. When adjusted for age, sex, BMI, ASA classification, CCI score, primary surgeon grade and patella resurfacing, the OKS score was significantly higher in the RA-TKA group by 2.3 points (*p* = 0.041). The EQ-5D-5L index was similar between the CO-TKA group (median = 0.92; 0.86–1.00) and the RA-TKA group (median = 0.96; 0.91–1.00), even after adjustment (*p* = 0.335). Of interest, the median EQ-VAS was higher in the RA-TKA group (median = 82.5; 75.0–95.0) compared to the CO-TKA group (median = 75.0; 65.0–85.0). Adjusting for age, sex, BMI, ASA classification, CCI score, primary surgeon grade and patella resurfacing, the EQ-VAS score was higher by 10.8 points in the RA-TKA group (*p* = 0.037).

## Discussion

Bilateral simultaneous RA-TKA reduced the odds of inpatient complications by 79% without an increase in the risk of 12-month mortality, revision surgery or reoperations when compared with CO-TKA. We observed fewer patients with severe anaemia requiring blood transfusions in the RA-TKA group. This may be due to the elimination of intramedullary jigs when performing RA-TKA [24]. Furthermore, the improved soft tissue handling afforded by the semi-active haptic robotic system utilised at our institution may have also resulted in earlier mobilisation, which in turn may have reduced the frequency of early postoperative venous thromboembolic events [25]. Our mortality and complication results are similar to those of Cohen et al. [14]. The lack of difference in revision or reoperation rates is unsurprising, given the limited follow-up period in the RA-TKA group. Although our study is the first to describe this in bilateral simultaneous TKA, it mirrors the findings by the Australian Orthopaedic Association National Joint Replacement Registry [26]. Improvement in alignment and component positioning through the use of RA-TKA is intended to reduce implant wear rates [27, 28]. Thus, the revision benefits would likely only be realised in long-term studies.

When adjusted for patient and surgical factors, HLOS was shorter in the RA-TKA by 1.1 days. This outcome was also reported by Cohen et al. [14]. Nonetheless, the HLOS for bilateral simultaneous TKA at our institution was slightly longer than that reported by the same authors, representing an avenue for future improvement. Furthermore, in contrast with the study by Masilamani et al. [16], we found no difference in surgical time between RA-TKA and CO-TKA. This may be due to the relatively low volume of bilateral simultaneous TKAs performed at our institution. Functional outcomes in our patient cohort after 12 months from their TKA were promising. The OKS in the RA-TKA group was significantly higher than the CO-TKA group but did not exceed the minimum important difference [23]. Similarly, we found no difference in the EQ-5D-5L index. It is likely that the lack of an observed difference may be due to the “ceiling effect” of these scores. Of the surveys available for analysis, 24.7% of patients had the ceiling OKS and 36.1% had the ceiling EQ-5D-5L index. Further studies should utilise questionnaires developed using item response theory and/ or computer adaptive testing to overcome this limitation [29, 30]. We did, however, demonstrate a significant improvement in the EQ-VAS score of 10.8 points, after adjusting for patient factors and surgical technique. This increase is substantially greater than the minimum important difference reported in previous studies [31, 32].

The key strength of this study is the utilisation of prospectively collected data within a single institution, which reduces the risk of recall bias. Nevertheless, several limitations need to be addressed. Firstly, we did not have a uniform policy for patient selection, and the decision to proceed with bilateral simultaneous TKA was based entirely on surgeon and patient preference. However, analysis of patient demographics and relevant comorbidities did not reveal any significant differences in patient selection that would substantially alter the outcome. Secondly, the use of tourniquet and patella resurfacing was not standardised in this study. These were considered in the analysis and adjusted for where appropriate. It is possible that our outcomes may be inadvertently influenced by other factors that were unaccounted for in our adjusted analysis, and therefore, randomised controlled studies are required. Thirdly, as mentioned previously, the measurement of our functional outcomes encountered substantial floor and ceiling effects. Fourth, patient enrolment in this study spanned for more than a decade. The prolonged recruitment period may have reduced between-group comparability and thus potentially undermining the reliability of observed differences. Fifth, in the RA-TKA group, we did not record the turnaround time, as it was felt to be immaterial as an outcome. Additionally, there was a relatively low volume of bilateral simultaneous TKAs performed at our institution throughout the study period, with a limited number of patients in each group. This limits the power and generalisability of our findings, and thus, our results should be interpreted with caution. Lastly, we did not compare our findings with bilateral staged or staggered RA-TKA, representing an exciting avenue for future research.

In conclusion, our study found that bilateral simultaneous RA-TKA was associated with reduced HLOS and lower complication rates, without any significant differences on 12-month mortality, revision, or reoperation risks compared with CO-TKA. Importantly, the frequency of postoperative symptomatic anaemia requiring blood transfusion and venous thromboembolism was lower in the RA-TKA group. There was no difference in surgical duration between the two groups, nor clinically relevant improvement in the OKS or EQ-5D-5L index. Nonetheless, there was a clinically and statistically significant improvement in the EQ-VAS score. Bilateral simultaneous RA-TKA thus provide some benefits over CO-TKA, although multi-armed randomised controlled studies are required to confirm the findings of this study and to determine whether there is a benefit over bilateral staged RA-TKA.

## Data Availability

The datasets generated and/or analysed during the current study are not publicly available due conditions imposed by our ethics committee, in the interest of patient privacy, but are available from the corresponding author on reasonable request (subject to further approval from our ethics committee).
